# Standardized assessment, information, and networking technologies (SAINTs): lessons from three decades of development and testing

**DOI:** 10.1007/s11136-020-02528-z

**Published:** 2020-05-25

**Authors:** John H. Wasson

**Affiliations:** Dartmouth - Geisel School of Medicine, Lebanon, NH 03756 USA

**Keywords:** Patient engagement, Risk assessment, Health confidence, What Matters Index, Howsyourhealth.org, Guided healthcare

## Abstract

**Purpose:**

To rectify the significant mismatch observed between what matters to patients and what clinicians know, our research group developed a standardized assessment, information, and networking technology (SAINT).

**Methods:**

Controlled trials and field tests involving more than 230,000 adults identified characteristics of a successful SAINT—www.HowsYourHealth.org—for primary care and community settings.

**Results:**

Evidence supports SAINT effectiveness when the SAINT has a simple design that provides a service to patients and explicitly engages them in an information and communication network with their clinicians. This service orientation requires that an effective SAINT deliver easily interpretable patient reports that immediately guide provider actions. For example, our SAINT tracks patient-reported confidence that they can self-manage health problems, and providers can immediately act on patients’ verbatim descriptions of what they want or need to become more health confident. This information also supports current and future resource planning, and thereby fulfills another characteristic of a successful SAINT: contributing to health care reliability. Lastly, SAINTs must manage or evade the “C-monsters,” powerful obstacles to implementation that largely revolve around control and commercialism. Responses from more than 10,000 adult patients with diabetes illustrate how a successful SAINT offers a standard and expedient guide to managing each patient’s concerns and adjusting health services to better meet the needs of any large patient population.

**Conclusion:**

Technologies that evolve to include the characteristics described here will deliver more effective tools for patients, providers, payers, and policymakers and give patients control over sharing their data with those who need it in real time.

## Background and methods

Health care providers have historically relied on patient statements to diagnose conditions and direct treatments. Since the advent of formal health care quality assessment in the 1960s [1], standardized patient-reported measures have become a tool for explicitly enumerating needs and documenting providers’ progress toward meeting those needs [2].

In the 1980s, our practice-based research network documented a significant mismatch between patients’ reports of their physical and emotional problems and what clinicians knew about those problems, if they knew anything at all [3, 4]. The implications of this mismatch for patient health and satisfaction with care provoked us to identify eight single-item measures of patient physical, emotional, and social function that could be used both to guide service and to monitor change [5]. The World Organization of Colleges, Academies and Academic Associations of General Practitioners/Family Physicians quickly adopted these measures, called the Dartmouth COOP Functional Assessment Charts, and translated them for worldwide use [6].

By the early 1990s, Rubenstein et al. had conducted a controlled trial to test whether a complex, multi-variable measure of patient function—the SF-36—could “be used by physicians in practice to help improve their patients' outcomes” [7, 8]. Concurrently, our research group conducted a controlled trial of the Dartmouth COOP Charts to assess the short-term effects of that approach on the process of care and patient satisfaction [9]. The SF-36 study found “no significant differences between experimental and control group patients at exit from the study on any functional status or health outcome measure” and concluded that a “more powerful intervention … is needed to help office-based internists improve patient outcomes” [8]. In the Dartmouth COOP Chart study, we found a small improvement in satisfaction with pain management, yet no significant impacts on patient or population health.

These early studies indicated the need for both explicit information that will be useful to care providers and service feedback loops between providers and patients. Over the next several decades, we therefore tackled the challenge of designing efficient feedback systems to enhance the impact of our assessment measures by alerting clinicians to patients’ self-reported needs—the necessary first step for helping them. As our assessment, information, and networking technology evolved, clinicians in our research group field-tested the various adaptations.

In a controlled study conducted in 1999, we compared responses from 832 elderly patients who merely received the self-assessment survey with responses from 819 intervention patients who received the survey in conjunction with automated need-specific instructions, and whose responses were automatically relayed to their physicians [10]. The patients in the intervention group felt their physicians were better informed of their needs and reported greater understanding of their health risks, as well as help with limitations in daily activities, emotional issues, and social support. Over the 2-year study period, eight of the 11 intervention practices improved their relative standing with regard to how their patients judged them. Only one of the 11 usual care practices showed this improvement.

Another controlled trial in 2006 tested web-based messaging between 47 physicians and 644 adult patients with pain and emotional problems [11]. The results of this study showed sustained improvement in patients’ pain and function at 6 months when our computerized system was combined with a problem-solving intervention supported by a nurse educator.

In summary, the Dartmouth COOP Charts’ simple measures of what matters became the starting point for a standardized assessment, information, and networking technology (SAINT). To date, more than 200,000 patients aged 19–69, 30,000 aged 70+, and 10,000 adolescents and children have used versions of our SAINT, www.HowsYourHealth.org, for guiding clinician action on needs that matter and improving patient health and satisfaction [12]. Thus, the following observations of the characteristics of an effective SAINT are based on decades of field tests and controlled trials and the responses of many users.

## Characteristics of an effective SAINT

### Easy to use: provides a service that is simple and cheap

To evaluate patient function, population health, or practice performance, and to allocate reimbursements to clinicians and health care systems, policymakers and payers have adopted many multi-item patient-reported instruments, such as the SF-36, the more recent PROMIS-29 and PROMIS-10, and many versions of CAHPs [13–16].

We designed our SAINT as a simple automated feedback system for the front lines of health care delivery, where patients and clinicians immediately co-produce a service. We emphasized single measures to improve efficiency, encourage participation, and stimulate action, and we showed that single queries of patients are both appropriate and more cost-effective as substitutes for several multi-item measures in evaluations of: practice quality (compared to CAHPs) [17], domains of patient function (compared to SF-36) [5], and patient engagement (compared to six measures for confident self-management contained in a Patient Activation Measure) [18].

Another consideration critical to the design of an effective SAINT is that clinicians have to operate on a lean business model and expect low direct and indirect costs for front-line users. SAINTs are commodities that must compete with hundreds of thousands of health care applications, and in the USA, the measurement industry is increasingly considered a source of significant health care waste, such that high pricing is not likely to be tolerated [19–22]. Fortunately, as our SAINT evolved, the internet came to provide a very inexpensive alternative to our earlier distribution methods, which had relied on scannable paper bubble forms, bar codes, and touchscreen kiosks. The web-based SAINT has allowed us to make it available to any interested health care providers at no cost through www.HowsYourHealth.org. The internet has also allowed schools and municipalities to disseminate our SAINT widely without cost [12, 23–25].

Thus, a successful SAINT must aim to serve, not just survey. Our SAINT was developed for people aged two and older and includes tools that support general problem-solving and decision-making, as well as special versions for homebound patients and those in the hospital [26, 27]. Variations of the SAINT have also been developed to comply with different types of regulatory requirements, including those of the Center for Medicare Services and state Medicaid authorities, as detailed at https://howsyourhealth.org/static/HYHModifications.pdf.

### Guides action: reduces clinician guesswork about what matters to patients

Measurement systems designed principally for retrospective population analyses are of little use to health care providers, who need prospective guidance for individual patients. Therefore, a service-oriented SAINT must enable timely, easily interpretable patient reporting that guides action.

As more and more people used our SAINT, health confidence emerged as one of the measures that mattered most to most patients [28], and exemplified a measure that could guide action for providers, patients, and community services. Health confidence is a single-item measure of overlapping concepts of self-management capacity, engagement, self-efficacy, and activation [29–31]. People who are designated as health-activated, or those who simply report confidence that they understand and can manage most of their health concerns, use fewer costly health care services [18, 31–33]. When a practice routinely measures and responds to health confidence, costly care use seems to decrease [34, 35], and many other patient-reported outcomes such as healthy eating and risk reduction are associated with health confidence [31].

Health confidence is also a good indicator of effective communication between patients and clinicians. For example, after adjusting for baseline characteristics, more than two thirds of patients who became more confident over time also reported that their clinicians were aware of and provided good education about emotional problems [36]. Another example showed a strong correlation between the health confidence of patients with asthma, diabetes, heart disease, high cholesterol, or hypertension and the extent to which clinicians allowed these patients time to ask questions, encouraged their involvement in decision-making, and explained care in language that was easy to understand [18].

Health confidence is undermined by pain and emotional problems [36]. With this knowledge, we investigated the possibility that a few measures, including health confidence, pain and emotional problems, and perceptions of adverse medication effects, might be more clinically useful than algorithm-based predictions generated from administrative data. We found that only five measures, called the What Matters Index (WMI), could together forecast future costly care, immediately guide care for most patients, and were a suitable proxy for patient quality of life [37–39]. Table 1 lists the WMI questions and possible actions a medical assistant might take in response to patient answers to each measure.

**Table 1:** The What Matters Index and recommended actions based on responses

Patient-reported measure	Examples of medical assistant actions for concerns or problems
Insufficient health confidence	
How confident are you that you can manage and control most of your health problems? *(Not very confident or somewhat confident scored as 1, versus very confident scored as a zero)*	You indicated that you are only somewhat or not very confident to manage and control your health problems. The health problems you find most difficult to manage and control are: ––––– What would it take to increase your health confidence to better manage and control these problems? –––––
Pain	
During the past 4 weeks how much bodily pain have you generally had? *(Extreme or moderate pain scored as 1, versus none, very mild or mild scored as a zero)*	Is your doctor or nurse aware of pain or emotional problem(s)? Yes No or not sure → referral to health professional How much is (are) your pain/emotional problem(s) making it difficult for you to be confident about managing your health?
Emotions	
During the past 4 weeks how much have you been bothered by emotional problems such as feeling anxious, irritable, depressed, or sad? *(Extremely or quite a bit scored as 1, versus not at all, a little or somewhat scored as a zero)*	Making it very difficult Making it somewhat difficult Not much impact
Polypharmacy	
How many prescription medicines are you taking more than 3 days a week? *(More than five scored as 1, versus 5 or less scored as a zero)*	The last time a health professional reviewed them with the patient or caregiver was more than 2 months (or not at all) → referral to a health professional
Adverse effects from medicines	
Do you think any of your pills are making you sick? *(Yes or maybe scored as 1, versus no scored as a zero)*	

The "What Matters Index" is the sum of the five binary scores with an index of zero meaning lowest reported problems and an index of five meaning highest reported problems

Reprinted with permission [37]

**Fig. 1:**
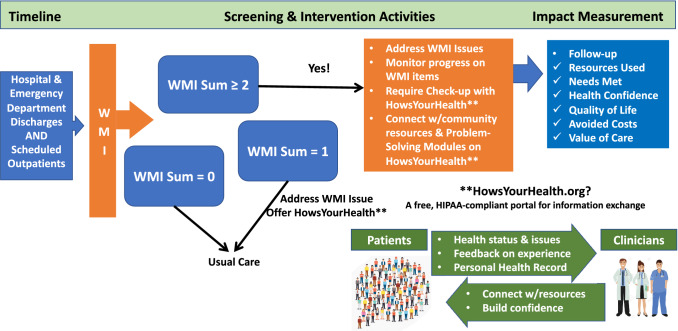
The What Matters Index as an effective SAINT: an immediate guide for care to reduce risk for costly emergency or hospital use

Although our SAINT includes health confidence and the WMI in a more comprehensive platform that connects patients, providers, and community-based support services, a SAINT could also be effective using only health confidence or the WMI. A very brief SAINT could be offered on paper or a handheld device as a population-based screener to guide care and serve as a gateway for further inquiry. Figure 1 illustrates the logic and flow of WMI screening in our current version of www.HowsYourHealth.org.

### Guides resources: contributes to health care reliability and resource planning

Absent adequate preparation, merely knowing what matters to each patient is not a guarantee that a small office practice or a larger health system will have exactly the resources most patients want and need exactly when and how the patients want and need them. Resource planning, as this preparatory activity is often called, has been the subject of decades of health services research. For example, Wagner identified several essential properties of successful health systems, founded on the understanding that effective chronic care management requires productive interactions between engaged patients and prepared, proactive providers [40–42].

Building on that work, our research group described how clinical microsystems can apply the model’s principles at the practice level. Our work showed that effective clinical microsystems must allocate resources based on the measurement and analysis of what matters to patients, always aiming to maximize the productivity of each patient interaction—no matter how brief—with planned, proactive care [43]. Thus, a WMI-based SAINT supports productive interactions that immediately serve patient needs. By standardizing the interaction, automating the information exchange, reducing clinician guesswork about what matters, and guiding subsequent clinical responses, this SAINT facilitates effective care and also signals where and how to focus resources. We have shown that this patient-centric approach, when extended to all patients, is associated with improved service quality [34, 35].

Specifically, before an office visit, our SAINT asks patients who are not health confident what they want or need to become more health confident, so that at the time of the office visit, staff will already know what resources are required to meet each patient’s needs. As more and more patients use this SAINT, their aggregated data indicate the sum of resources required to meet most patient needs most of the time. We have used a similar process to identify causes of unnecessary or harmful care [44], and those data guide resource planning away from unproductive or even counterproductive investments. The quality-of-care information produced by our SAINT is also increasingly accepted by those who pay for, certify, and regulate health care [45, 46].

This approach stands in stark contrast to current health resource planning in the USA, where fragmented and inconsistent health care, delivered at multiple points of service, erodes reliability for both the affluent and the poor [47]. While hospitals and practices are major contributors to this problem, service fragmentation extends to community resources as well. For example, a physician member of our research group tallied health care-related contacts for 386 older persons living in a community of 2600 inhabitants and discovered more than 30 stand-alone organizations representing generalist care, specialty care, nursing care, and social services. Without a tool that measures and analyzes patients’ self-reported resource needs, none of these organizations can predict what resources they should have available, let alone coordinate with each other to reduce waste and maximize efficiency.

### Heeds the C-monsters: content, confidentiality, control, consent, culture, cost, copyright, coding, and commercialism

During the evolution of our SAINT, feedback from patients, providers, payers, and policymakers pointed to certain hazards that will limit a SAINT’s value, dissemination, and sustainability, and will ultimately result in failure to improve patient health and satisfaction.

First, the SAINT must get the goal right: The SAINT’s content should support service, not just measurement or reimbursement. Unless the patient and clinician see immediate benefit, the SAINT is likely to fail, regardless of its elegant appearance and psychometrics.

From the patient’s perspective, the SAINT must also support confidentiality, control, and consent. Our SAINT assures these with a privacy design that assumes patients expect absolute control over their identifying information, and do not want to be subjected to advertising or conflicts of interest. The European Union recently codified these standards in the General Data Protection Regulation. However, in the USA, a SAINT may be accessible only through a hospital-sponsored portal that links the responses to a medical record, and people are often reluctant to give up personal identifiers just to complete a questionnaire of unknown content and purpose. For these reasons, our SAINT—www.HowsYourHealth.org—allows completion before collecting identifiers, and offers patients a personal, portable health plan with no identifiers. To support patients’ cultural needs, we have found that translation into another language and back to the original before dissemination can identify inappropriate interpretations and unsuitable cultural content.

From the clinician’s perspective, a SAINT must avoid the high costs associated with the hazards of copyright enforcement, proprietary coding, and commercialism. For these reasons, we have always made our SAINT freely available and adaptable for research and practice without charge and only request that the copyright source be listed. Proprietary coding often obstructs customization and communication, and commercialization, exemplified by the many hundreds of competing, incompatible electronic medical records in the USA, obstructs co-production of care and resource planning among clinical settings.

Based on observations over decades of experience, Table 2 suggests characteristics that are likely to enhance a SAINT’s value, dissemination, and sustainability … and evade the “C-monsters.”

**Table 2:** Suggestions for enhancing SAINT value, dissemination, and sustainability

√ or	If no √, this is a threat	Explanation
Enhancing SAINT value		
	Goal: patient quality of life	Patients’ engagement is highest when the SAINT generates service for what matters to them; measurements for comparison and process adherence are secondary
	Stakeholder: the patient-clinician dyad	Although payers, purchasers, and policymakers can be partners, front-line engagement of both the patient and clinician is critical for success and co-production of best care
	Focus: data guides action	Service, not just survey: multi-item, psychometrically elegant data are often not intended for action
	Latency: short	Long latency and retrospective data undermine accuracy and action
	Stratification: offer a registry	Data are used to sort groups of patients for subsequent interventions
	Customizable: for practice	Facilitates adaption to and adoption in multiple settings
	Portable: for patient	Enhances recall, useful communication with others, updating and monitoring
	Improving: summaries	Ongoing assessment of clinician and practice performance
	Behavioral: automates	Minimizes variation and effort needed to implement initial behavioral interventions
	Links: community and other	Minimize to only highly relevant links
	Online consent: for follow-up	Useful to the practice or clinician for longitudinal quality and research projects
Enhancing SAINT dissemination		
	SAINT product design: simple	Start with the core functions and basic display; continuously test variations
	Process fit: timing	Reduce initial patient implementation target by half and double the time
	Helper: patient volunteers?	Patients of practice may volunteer to assist; build on small successes
Enhancing SAINT sustainability		
	Engineer at outset: small burn	Burn refers to the amount of money needed to design and maintain
	C-Monsters: be vigilant	Watch out for the most common destructive forces: high burn rates and loss of control
C-Monsters—adversely influence value, dissemination and sustainability		
	Content	Minimize medical advice to decrease liability; there are few insurers who cover SAINTs
	Consent for identifier (privacy)	Offer best content regardless of patient identifier; postpone identifier to later in process
	Control	Business models and regulations of a country impact who and how a SAINT is controlled; consideration of the short- and long-term consequences is necessary
	Culture	Translation of measures into another language and back to the original before widespread dissemination to mitigate obscure interpretations or unsuitable cultural content
	Copyrighted materials	High value SAINTs should allow fair use; avoid expensive and restrictive licenses
	Computer coding	Open source software provides common, time-tested code to minimize maintenance
	Confusing commercialism: e.g. electronic Medical record	Proprietary interests and inflexibility impede data import; priorities should support patient/clinician coproduction of care and a patient-controlled portable health plan/record
	Certification	Adopt the highest ethical standards; then estimate the added value of certification

## Principles into practice and policy: an illustration for patients with a chronic condition

**Fig. 2:**
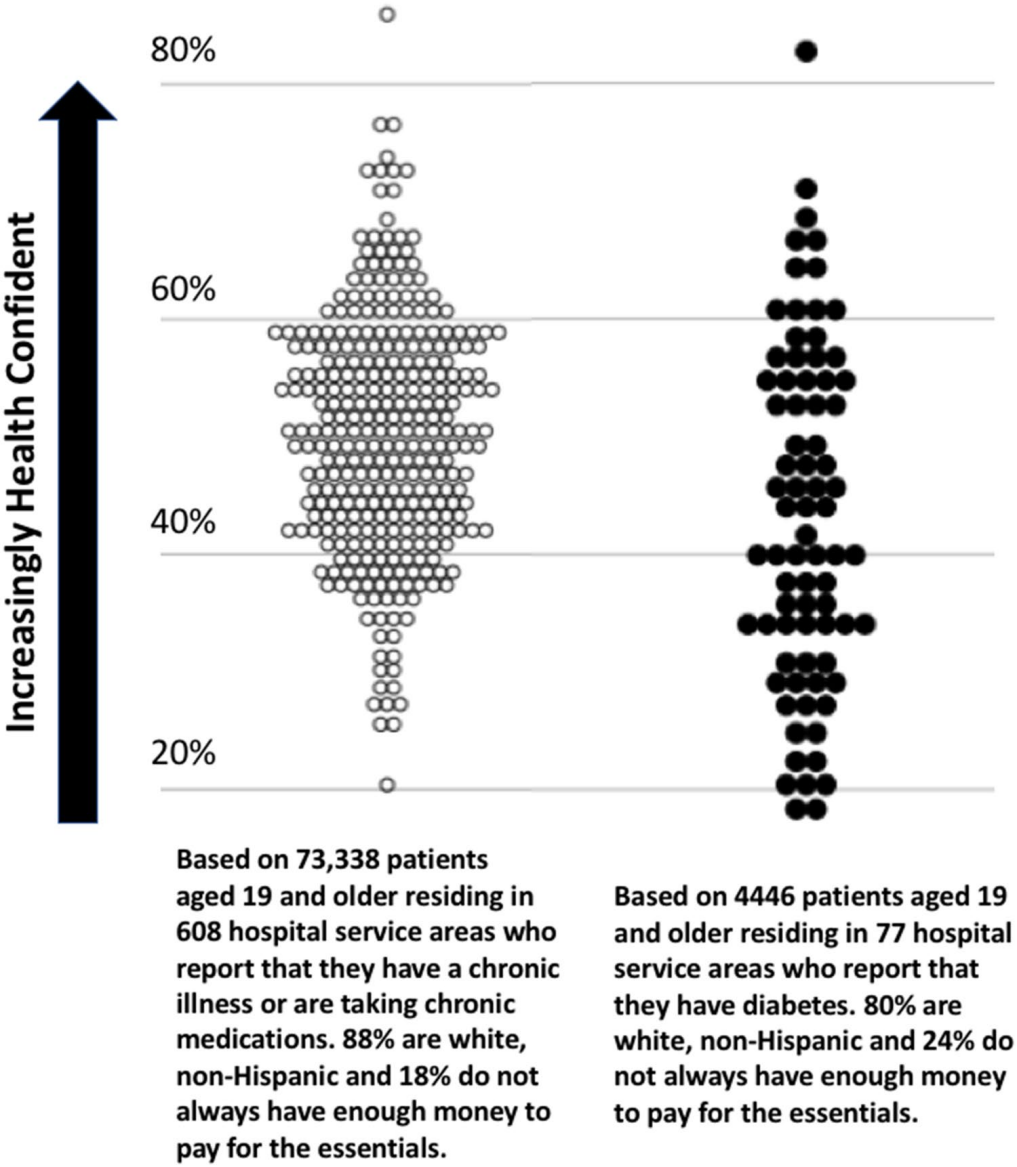
Percent of patients in US hospital service areas (HSAs) reporting they are very confident that they understand and can manage most of their health problems; data from HowsYourHealth.org were matched with HSAs defined by the Dartmouth Atlas of Health Care [48]

This section shows how our SAINT leverages the simple measures of health confidence and the WMI to guide care toward what matters to patients and to improve health care reliability by standardizing service. Figure 2 illustrates the wide variation in health confidence levels across hospital service areas (HSAs) in the USA, for 73,338 adult patients with any chronic condition in 608 HSAs, at left, and 4446 adult patients with diabetes in 77 HSAs, at right. The health confidence data were drawn from HowsYourHealth.org and matched with geographic HSAs based on ZIP codes aggregated by the Dartmouth Atlas of Health Care [48]. Analyses based on the presence or absence of poverty, pain, and/or emotional problems shift the mean and median of the data in the figure but do not meaningfully lessen the population-level variation in health confidence across these HSAs.

**Fig. 3:**
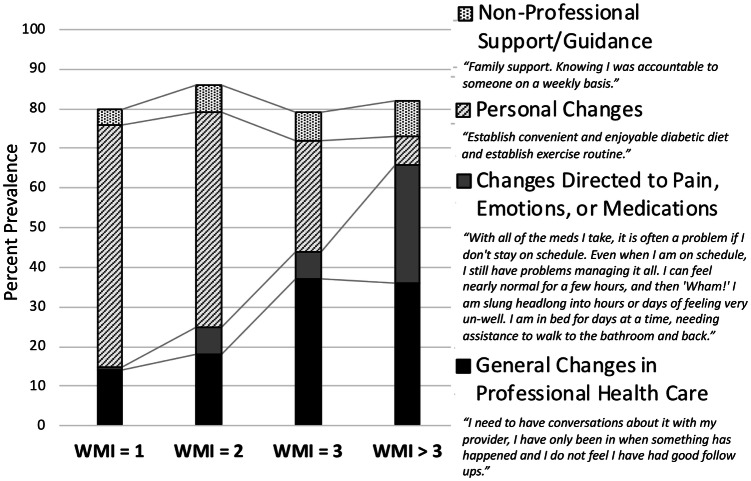
Patients with diabetes describe what they need to attain greater health confidence: Based on verbatim responses, exemplified at right, of more than 600 patients collected via HowsYourHealth.org since 2017, excluding “don’t know” or uninterpretable responses. The *WMI* (What Matters Index) is the sum of five patient-reported problems and concerns: **a** insufficient confidence to self-manage health problems, **b** pain, **c** bothersome emotions, **d** polypharmacy, and **e** adverse medication effects

Unreliable delivery of health care services has long been recognized as the cause for undesirable variation, for which quality management via standardization is a potent corrective [49]. To reduce variation, practitioners have used our SAINT to proactively ask every patient with low health confidence what would be most likely to help them gain confidence. As an example, Fig. 3 summarizes the relationship between what patients with diabetes said they needed to become more health confident, in relation to their WMI scores. In this example, respondents with higher WMI scores were more likely to identify a need for professional assistance, and less likely to believe that changes in their personal behavior would improve their health confidence. In addition to understanding what matters to each patient now, the practice can plan for future service demands knowing both the distribution of its patients’ WMI scores and the verbatim responses of many respondents.

Absent a standard report from each patient about what matters, both generalist and specialist clinicians confront the dilemma shown in Table 3. This table summarizes self-reports on a range of topics from more than 10,000 patients with diabetes, as well as these patients’ diagnoses, risks, and service use. The table sorts the patients by their WMI scores, and patients with higher WMI scores can clearly be seen to report far more extensive symptoms, limitations, and concerns, and less engagement in self-management. These patients are also much more likely to be burdened by co-occurring health conditions and to use potentially avoidable costly care.

**Table 3:** Self-report from diabetic patients illustrating how a What Matters Index guides care and is an expedient proxy for what else might matter

	Sum of What Matter Index is			
	0	1	2	3
Number	2205	3197	2491	2327
WMI guidelines indicated				
Not health confident	NA	63	80	94
Bothersome pain	NA	8	32	75
Bothersome emotional problems	NA	3	14	47
Medications may be causing illness	NA	7	36	68
Poly-pharmacy (more than 5 medications/day)	NA	21	42	73
What else might matter	**↓**	**↓**	**↓**	**↓**
Examples of self-management engagement				
Has received good explanation for chronic condition(s)	82	68	56	43
Almost always checking and controlling blood sugar	68	56	50	42
Checks blood pressure regularly	70	59	54	46
Exercises at least three times a week	50	30	24	14
Usually eats healthy meals	76	63	53	34
Examples of major limitations, symptoms and concerns				
Poverty: does not often have money for everyday needs	12	20	33	49
Very limited social support	7	11	16	34
Very limited social activities	1	2	9	32
Very limited daily activities	1	4	10	41
Very limited physical capacity	5	11	14	29
Significant sleep limitations	7	17	28	51
Concern about violence and abuse	3	5	7	15
Examples of common reimbursable foci for clinical care				
Additional diagnoses				
Hypertension	53	62	71	79
Arthritis	20	28	41	62
Respiratory	12	15	22	37
Atherosclerosis	8	16	23	40
Risks				
BMI > 30 (obese)	45	54	62	71
Previous recalled systolic blood pressure > 150	18	20	26	35
Previous recalled blood sugar > 140 (7.8 I.U.)	23	33	39	49
Current smoker	10	12	18	30
Costly health care and care quality				
Recent emergency department or hospital use	13	19	28	49
Any of emergency or hospital uses possibly avoidable	17	31	44	56
Any healthcare-related harms during previous year	0.6	0.9	1.6	5.0
Continuous care from a doctor or nurse	88	86	83	80
Using a specialist	38	44	51	59
If using a generalist and a Specialist, one is in charge	89	85	81	72
Easy access	67	49	39	29
Receiving exactly the care needed	59	40	31	18

Numbers in the table body are a percentage of the respondents in each column

Wording and cutpoints for these patient-reported measures are available at https://howsyourhealth.org/html/adult_survey.pdf

Thus, the conundrum facing any general or specialty care provider: During a brief face-to-face visit, often interrupted for documentation and billing activities, which of the many symptoms, illnesses, functional limitations, and aggravating lifestyle challenges should be the focus?

Historically, it was assumed that health care professionals could rely on clinical judgment to determine the most important focus and prescribe an appropriate treatment. However, clinicians can easily come to different conclusions based on how they interpret information and the contexts in which they work, including whether they provide specialty or generalist care, and in what setting. When deference to professional opinion is the predominant strategy, unreliable and ineffective care is often the result [50].

In recent years, payers and policymakers have promoted algorithm-based prediction instruments that use administrative and medical record data to identify very short lists of patients at risk for costly care, and have then incentivized clinicians to direct more time and services to these patients. A typical algorithm would categorize as high risk for costly care about 10% of the patients in Table 3 based on criteria of an additional diagnosis of serious atherosclerotic cardiovascular disease and a recent hospital admission or emergency department use.

However, predictive analytics and “hotspotting” strategies are proving to be inaccurate, cost-ineffective, and unethical because they direct resources away from the many patients not designated at-risk who are in fact destined to need costly care [38, 50]. The 10% of patients identified by those approaches will have a plethora of the issues listed in Table 3, and faced with this complexity, generalist and specialist clinicians justifiably fall back on their highly variable clinical judgments, often focusing on “sugar control” or other narrowly circumscribed clinical parameters. However, the selected focus is seldom, if ever, the only important challenge and may be subordinate to problems that impose greater burdens on these patients and the health system.

In contrast to the limitations of targeting a few outlier patients at risk for costly care based on old data, our SAINT’s WMI provides a timely, easy-to-interpret, actionable, and reliable foundation for predicting risk and organizing care, both within a practice and throughout a service area, with progress on any of the WMI measures likely to mitigate many associated problems. For example, when patients report that they are not health confident, the software asks them what they believe will be most helpful to improve it, and then sends the verbatim patient response to the clinician as part of a summary of the patient’s responses to every WMI item.

Consider two clinics that provide care to only patients with diabetes. Based on a sample of 30 patients in each clinic who complete the WMI, Clinic A recognizes that 70% of its patients have a WMI of 1 or less, whereas Clinic B learns that 70% have a WMI of two or more. From the information in Table 3, many patients in both clinics will require assistance to become more health confident. However, Clinic B will need to plan more resources to enhance its vigilance for adverse impacts of medications and support for the management of pain and emotional problems. The higher prevalence of patient poverty and social isolation presents an additional resource challenge for Clinic B.

In summary, this illustration calls to mind a useful analogy: that the diagnostic labels we give each patient are merely suitcases containing a jumble of symptoms, associated illnesses, aggravating lifestyle challenges, health-related concerns, functional limitations, and social factors. The WMI provides a standard and expedient handle for a generalist or specialist to move each patient’s suitcase toward the patient’s desired destination. Different diagnostic suitcases can use the same WMI handle.

## Conclusion

This report summarizes lessons from three decades of using a standardized assessment, information, and networking technology (SAINT). For patients with chronic conditions, the evidence supports SAINT effectiveness at improving patient health and satisfaction when the technology immediately serves patients and engages them and their clinicians in the co-production of better care.

We have emphasized the advantages of a What Matters Index (WMI) as a parsimonious starting point for almost any SAINT. The WMI has no direct cost and is unambiguous, highly accessible, and strongly correlated with patient-reported quality of life. The WMI has also proved reliable in predicting future costly care for poor and not-poor patients with and without chronic conditions [38], and the reduced variance in interpretation facilitates resource planning and thereby maximizes value and reliability. Thus, generalist and specialist clinicians who use a SAINT that contains the WMI are likely to avoid common obstacles to the co-production of high-quality health care [51].

This report’s focus on the WMI raises a legitimate concern about the inclusion or exclusion of other patient-reported measures or indices derived from a combination of measures. Because of the heterogeneity in patients’ needs, resource availability, and health workers’ responses, a SAINT is unlikely to have the same beneficial impact in all situations. Therefore, an effective SAINT must have a highly adaptable design to add or omit measures when they are needed for specific subgroups of patients or research protocols. This report has described how our SAINT was designed for adaptability, and available evidence suggests that it is likely to be cost-effective for improving health care services and patient outcomes [10]. Our hypothesis is that the SAINT methodology and WMI described herein should be considered standards for comparison to other measures and methods.

In summary, with low and decreasing response rates to traditional survey techniques [52, 53], new tools and business models are needed to assess and deliver what matters to patients. Technologies that evolve to include the characteristics described here will deliver more effective and efficient tools for patients, providers, payers, and policymakers and give patients control over sharing their data with those who need it in real time. The WMI-based SAINT, www.HowsYourHealth.org, provides one broadly applicable and inexpensive strategy that reduces clinician guesswork regarding what matters to patients and facilitates resource planning to improve health care reliability. A medical maxim entreats us: “Listen to the patient; she is telling you the diagnosis.” Here, we add: Listen to a few measures that really matter to most patients; those measures are telling you what to do.

## Data Availability

Data from HowsYourHealth.org were used to illustrate how the principles described in this report can be applied to clinical practice and policy by documenting: (a) variation across hospital service areas (Fig. 2, *n* = 73,338), (b) the many problems reported by patients with diabetes (Table 3, *n* = 10,220), and (c) suggestions from patients with diabetes for increasing their health confidence (Fig. 3, *n* = 603). The data are available from the author upon email request (John.H.Wasson@dartmouth.edu). No personal patient identifiers are collected or stored by HowsYourHealth.org.
